# A national survey-based method for monitoring noncommunicable disease risk factors among adolescents aged 15–17 years across India^☆^^☆☆^

**DOI:** 10.1016/j.mex.2025.103535

**Published:** 2025-07-24

**Authors:** Kalpanapriya D, Sadanandam Vemula

**Affiliations:** Vellore Institute of Technology, India

**Keywords:** Alcohol consumption, Cross-sectional study, NCD risk factors, Prevalence, Tobacco use

## Abstract

The National Noncommunicable Disease Monitoring Survey (NNMS) was designed to generate nationally representative estimates of key NCD risk factors and contribute to India’s national framework for NCD surveillance. This article presents the methodology and key findings related to adolescents aged 15–17 years. The NNMS was a cross-sectional, community-based survey conducted across India during 2017–2018. It was implemented by the Indian Council of Medical Research (ICMR) and the National Centre for Disease Informatics and Research (NCDIR), in collaboration with ten reputed research institutes covering both urban and rural areas. Standardized tools and procedures were employed to ensure consistency and data quality across sites. The survey achieved a high overall response rate of 96.6 %, with participation from 1531 adolescents and 1402 households. Key findings indicate that 3.1 % (95 % CI: 2.0 %–4.7 %) of adolescents reported current daily tobacco use, while 25.2 % (95 % CI: 22.2 %–28.5 %) engaged in insufficient physical activity. Conversely, 74.8 % (95 % CI: 71.5 %–77.8 %) met recommended physical activity levels. Information on dietary behaviors and alcohol use was also collected. As the first nationally representative survey focusing on adolescent NCD risk factors, the NNMS provides crucial baseline data to support India’s NCD Action Plan and inform future prevention and control strategies at national and sub-national levels.

• This study presents the first nationally representative methodology to estimate NCD risk factors among Indian adolescents aged 15–17 years.
• Multistage, community-based sampling design and digital data collection protocol ensured high coverage and data quality across 27 states.
• The method is adaptable for state-level surveillance and supports national action plans for adolescent NCD prevention and control.

## Graphical abstract

**Figure:**
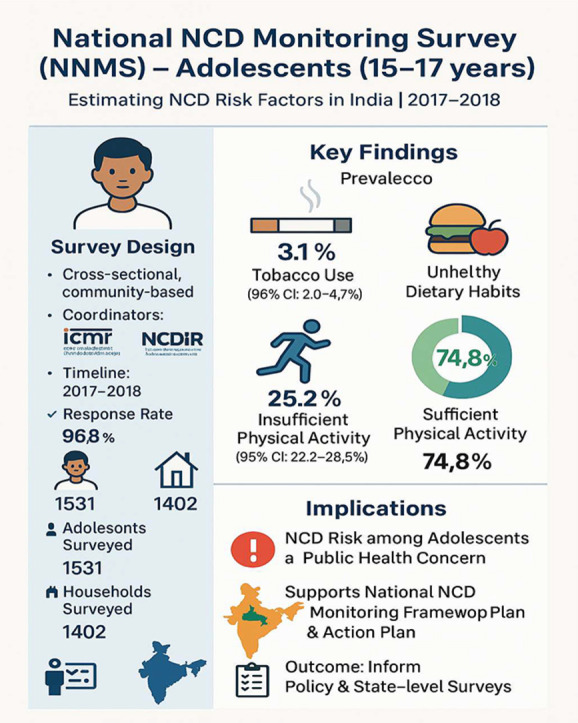

## Specifications table

**Table:** 

**Subject area**	Medicine and Dentistry
**More specific subject area**	Adolescent NCD Risk Surveillance
**Name of your method**	National NCD Risk Factor Monitoring Survey (NNMS)- A Cross-sectional Community-Based survey.
**Name and reference of original method**	Vemula S, Kalpanapriya D. Secondary Analysis of NCD Risk Factors in Northeast India: Insights from the National Cancer Registry Program. Indian J Comm Health. 2024;36(6):868–872. https://doi.org/10.47203/IJCH.2024.v36i06.021.
**Resource availability**	www.ncdirindia.org

## Background

Noncommunicable diseases (NCDs) are the leading cause of mortality worldwide [1]. Noncommunicable diseases account for 64.9 % of all fatalities in India, according to estimates. Cardiovascular diseases (CVDs) accounted for 27.4 % of all deaths among them [2]. One of the primary goals was to reduce the prevalence of high blood pressure in adults 18 years of age and over by 25 % relative to 2025 [3,4]. Infectious disorders, undernutrition, and diseases affecting mothers and children have all declined in India during the last 20 years as the burden of non-communicable diseases (NCDs) has increased [5]. In 2019, NCDs accounted for 65 % of all fatalities in the nation [6]. The main causes of these are the high prevalence of key preventable risk factors, which include smoking, drinking alcohol, having bad eating habits, not getting enough exercise, being overweight or obese, having high blood pressure, having diabetes, and having high cholesterol. Major noncommunicable illnesses (NCDs) as diabetes, cancer, stroke, and cardiovascular diseases (CVDs) are also influenced by their arrangement in persons.

Adolescents aged 15–17 years represent a critical developmental stage when long-term health behaviours—such as diet, physical activity, and substance use—are often established and can persist into adulthood, increasing future NCD risk. This subgroup is also transitioning toward greater independence in lifestyle choices, making it a pivotal point for early intervention. Additionally, they are logistically accessible through schools and community-based platforms, and are frequently prioritized in health monitoring frameworks due to feasibility and policy relevance. Monitoring NCD risk factors at this stage enables timely public health responses before clinical conditions emerge. Given India’s large adolescent population, focusing on this subgroup is both demographically strategic and aligned with global best practices, including WHO recommendations for early prevention. Their inclusion in the National Noncommunicable Disease Monitoring Survey (NNMS) 2017–18 was aimed at establishing a national baseline for key NCD risk factors among youth and informing adolescent-focused strategies within India’s public health programs.

According to the World Health Organization, smoking-related fatalities cause around 8 million deaths globally annually, making it a serious public health concern. However, alcohol misuse is responsible for 3 million fatalities worldwide each year, according to the WHO. Drinking alcohol raises the chance of developing mental and behavioral disorders, noncommunicable diseases like liver cirrhosis, a certain kind of cancer, and cardiovascular disorders. In addition, there was a decline in the prevalence of tobacco use, with reports of 39 % of men and 4 % of women using tobacco, compared to 45 % and 7 % of men and women in NFHS-4. Comparing the statistics from NFHS-5 and NFHS-4, respectively, showed that the current usage of alcohol by men decreased from 29 % to 19 % and by women from 1.2 % to 1 %. The incidence of non-communicable diseases has also sharply increased; in NFHS-5, the prevalence of hypertension in men and women has risen from 15 % and 11 % to 24 % and 21 %.

India is the second most populous country in the world, consisting of 28 states and 8 union territories (UTs). The six administrative zones/regions that comprise all Indian states and Union Territories are north, west, central, south, east, and northeast [7,8]. Every Indian area varies greatly in terms of its sociodemographic makeup, illness epidemiology, economic and social development, cultural and lifestyle habits, and sociodemographic profile. Moreover, regardless of a region's economic and health system characteristics, NCDs and the risk factors that are linked to them are becoming a significant issue [[9], [10], [11]]. Studying the regional-level profile of NCD risk factors is necessary in a nation this size and diversity. Plans and strategies for NCD prevention and control must consider these assessments. This study produced the prevalence and important NCD risk factors among adolescents aged 15–17 years using primary data from the National Noncommunicable Disease Monitoring Survey (2017–18).

## Method details

### Study design

The National NCD Monitoring Study (NNMS) is a cross-sectional study that was conducted from 2017 to 2018. The age range covered by the multistage cluster sample strategy was 15–69 years, 15–17 years for adolescents, and 18–69 years for adults. All of the accessible adolescents (15–17 years old) from the selected households were included in the survey. In collaboration with ten respectable implementing research institutes/organizations in urban and rural India, the National Centre for Disease Informatics and Research of the Indian Council of Medical Research performed the survey.

The surveillance methodology adopted in this study aligns with established frameworks such as the WHO STEPS approach [12], the Global School-based Student Health Survey (GSHS) [13], and the CDC Youth Risk Behavior Surveillance System (YRBSS) [14], while also drawing conceptual support from the Lancet Commission on Adolescent Health and Wellbeing [15]. The use of secondary data from the NNMS survey ensures national representativeness and consistency with India’s ongoing efforts to monitor adolescent noncommunicable disease (NCD) risk factors.

The NNMS 2017–18 was centrally coordinated by ICMR–National Centre for Disease Informatics and Research (ICMR–NCDIR), Bengaluru. ICMR–NCDIR was responsible for the overall survey design, development of survey tools and standard operating procedures (SOPs), obtaining ethical approvals, managing data harmonization, and overseeing quality assurance. Field implementation was carried out by ten partner institutions across different regions of India. AIIMS New Delhi conducted data collection in the Delhi region. ICMR–National Institute of Medical Statistics (ICMR–NIMS) supported the sampling methodology and developed the KISH grid for respondent selection. AIIMS Jodhpur was responsible for regional sampling coordination and field supervision. AIIMS Bhopal led efforts in retraining of field teams and real-time monitoring. ICMR–National Institute of Nutrition (ICMR–NIN), Hyderabad, provided expert guidance and training on anthropometric and biochemical measurement standardization. Assam Medical College carried out field data collection in the North-East region. SCTIMST, Thiruvananthapuram, developed and implemented the Trainer-of-Trainers (ToT) model and supported centralized quality assurance protocols. BJ Government Medical College, Pune, conducted field data collection and managed logistics coordination for the western region. ICMR–National Institute of Epidemiology (ICMR–NIE), Chennai, led data quality audits, provided training on epidemiological methods, and supported field protocol compliance. This multi-institutional collaboration ensured robust operational execution, regional coverage, methodological consistency, and national representativeness of the survey findings.

### Data collection

Field teams received structured training covering all key survey tasks, including household mapping and numbering, questionnaire administration, anthropometric and biomedical measurements (height, weight, waist circumference, blood pressure, and fasting blood glucose), equipment calibration, and biological sample handling. Data entry was conducted using electronic data capture tools such as tablets and mobile-based applications to ensure accuracy and efficiency. Ethical considerations, including informed consent and confidentiality protocols, were emphasized throughout the training. Each team comprised three multipurpose social workers (MSWs), with the most experienced member designated as the team leader. At least one MSW in every team was a woman, responsible for conducting anthropometric assessments and interviewing female respondents to ensure cultural appropriateness. The training also emphasized standardized measurement protocols and practical demonstrations to minimize inter-observer variability and ensure high data accuracy. To ensure data quality, field activities were supervised by regional investigators and centrally monitored by ICMR–NCDIR. A robust quality assurance framework was in place, involving routine supervisory visits, random spot checks, and systematic data validation procedures to maintain consistency and reliability throughout the survey.

### Sampling and allocation of sample size for adolescents aged 15-17 years

**Fig. 1:**
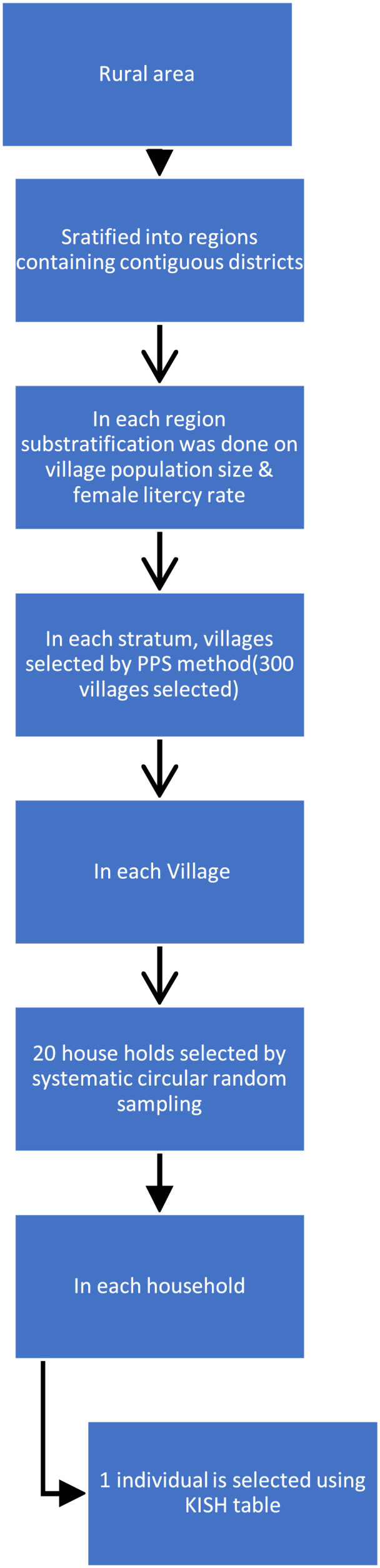
NNMS sampling design in rural areas.

The National NCD Monitoring Survey (NNMS) targeted study population included all adult individuals aged 15–69 year, the complete adolescent population (inclusive of both genders) aged 15–17 years, and residents of both rural and urban parts of the nation. A stratified multistage sample design was employed in the survey, encompassing 27 states in India. For adolescents (15–17 years old), the expected sample size was 1700 in total. For urban areas, the PSUs were wards, and for rural areas, they were villages. A random selection process was used to choose one Census Enumeration Block each ward. The sample frame consisted of all adults and adolescents combined, and it was split into four subgroups or strata: men and women, urban and rural (2 × 2). With an adult obesity prevalence of 9 %, a relative precision of 15 %, and a non-response rate of 15 %, the number of homes to be covered was estimated to be 12,000 (6000 in urban areas and 6000 in rural areas). The sampling frame in rural areas is shown in Fig. 1, whereas the sampling frame in urban areas is shown in the supplementary file Fig. 1.

Circular systematic sampling was used to choose 20 houses from each PSU. If one adolescent was chosen from each family, the sample size for adolescents would be 1440 based on the percentage of adolescents as 10 %–15 % from the Census 2011 and the assumption that 12 % of adolescents were accessible in the 12,000 selected households for adults. However, we covered all the available adolescents (15–17 years) in the selected household to reach the required sample size.

**Sample size**: There were a total of 1819 teenagers (15–17 years old) living in the 1402 participating families; 1643 of them took part in the survey while 176 did not because their homes were locked.

### Statistical analysis

We used IBM SPSS for Windows version 22.0 to clean the data. This step involved determining whether the variables were of the correct type (numerical or string), making sure that the variables were checked for duplicates, missing, conflicting, or illogical responses, coding for missing data, skip patterns, or unanswered questions, performing descriptive statistics, and using frequency tables to look for outliers. Using previously created analysis commands from sophisticated survey analysis, STATA 14.1 was used to analyze the data. As a gauge of accuracy for the estimated population parameters, the survey results have been displayed using descriptive statistics that include means and proportions with 95 % confidence intervals (CIs).

### Study tools

The survey was conducted using standard study instruments for adults [16], such as the WHO Stepwise Approach to Noncommunicable Disease Risk Factor Surveillance (WHO-STEPS) [17], the Integrated Disease Surveillance Project (IDSP)-NCD Risk Factor Survey [18], and GATS, India [19]. Eleven regional Indian languages were translated from English, the language used to create the learning materials. By adopting the forward-backward translation approach, the translated surveys underwent validation. An Android fine application called Open Data Kit was used to conduct study interviews on handheld tablets [20].

**Inclusion & Exclusion Criteria:** Adolescents in India, aged between 15–17 years are included in the study, all other age groups were excluded.

### Indicators and definitions

**Fig. 2:**
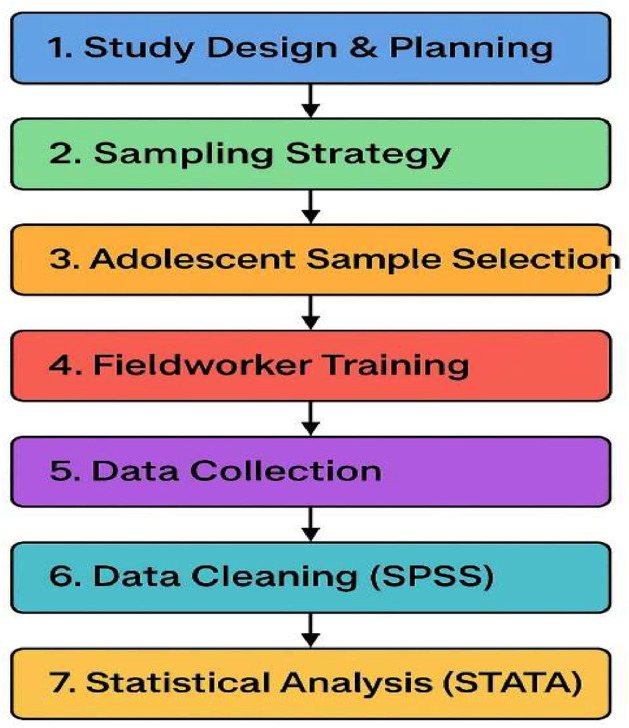
Methodological flowchart outlining the steps from study design to statistical analysis, including adolescent sample selection, fieldworker training, data collection, data cleaning (SPSS), and final analysis (STATA).

Survey information on behavioural risk factors and clustering risk factors and their standard definitions are mentioned in the supplementary file Table 2 [21]. A methodological flowchart outlining the steps from study design to statistical analysis is presented in Fig. 2.

## Method validation

All physical measurement instruments—specifically, Omron HEM 7120 digital blood pressure monitors and Gluco Spark glucometers—were calibrated both before and during field deployment. Calibration followed standard operating procedures based on manufacturer guidelines, and logs were maintained by field supervisors. Consistency in measurements was ensured through periodic cross-verification with reference devices, and any discrepancies were promptly corrected. To address inter-rater reliability, fieldworkers underwent structured training, including hands-on practice and periodic retraining sessions. Blood pressure readings were taken twice for each participant, and their average was used to improve precision. The survey instruments were adapted from internationally validated tools such as the WHO STEPS and Global School-based Student Health Survey (GSHS). Pilot testing was conducted to assess local relevance and ensure uniform tool administration. As the present study is based on secondary data from the nationally representative NNMS 2017–18 survey, internal reliability statistics such as Cronbach’s alpha were not computed independently. However, the original NNMS instruments were validated during their development, and their implementation followed standardized protocols, structured training, and pilot testing—ensuring high validity and reliability.

The percentage of adolescents (15–17 years) who responded was 93.2 %. There were a total of 1819 adolescents (15–17 years old) living in the 1402 participating families; 1643 of them took part in the survey, while 176 did not because their homes were locked. The survey was completed by 1531 adolescents in total, however, 112 of them declined halfway through or after first accepting it. 52.7 % of the respondents to the study who were between the ages of 15–17 year were females, and 47.3 % were boys. In the Public health care system as well as private primary care clinics have been surveyed system, a total of 335 district hospitals (DHs), 415 community health centers (CHCs), and 537 public primary care institutions that served the chosen PSUs were surveyed. In addition, 512 private primary care clinics within the same PSUs were questioned. 48.4 % of the population was male 51.6 % was female in urban areas, 46.4 % of the population was male and 53.6 % was female in rural areas. The response rates among the 15–17-year-old adolescents who responded were 90.1 % in urban areas and 95.9 % in rural regions based on where they lived. According to the poll, 94.2 % of adolescents completed formal education, with 97.3 % living in urban regions and 92.7 % in rural ones: 95.6 % of boys and 92.6 % of girls. A maximum of 79.3 % of adolescents between the ages of 15 and 17 reported attending high school (86.7 % in urban and 75.8 % in rural regions; 84.2 % of boys and 73.9 % of girls). 3.3 % of girls and 1.5 % of boys reported graduating, with 2.3 % of respondents residing in rural areas and 2.5 % in metropolitan regions.

## Prevalence of NCD risk factors

### Tobacco use

Just 3.1 % (95 % CI: 2.0 % to 4.7 %) of adolescents (15–17 years) said the current use of any form of tobacco products daily in the 12 months prior to the study. In both rural areas (3.6 %, 95 % CI: 2.3 % to 5.8 %) and among boys (6.5 %, 95 % CI: 3.9 % to 10.7 %), the proportion was higher. Roughly twice as many people (7.0 % (95 % CI: 5.4 % to 9.1 %)) said they had tried smoking or using smokeless tobacco in the past. The prevalence of using smokeless tobacco was higher than that of using smoked tobacco, at 2.8 % (95 % CI: 1.7 % to 4.3 %) compared to 0.2 % (95 % CI: 0.03 % to 0.8 %). Results indicated that males used smokeless tobacco more frequently (6 % 95 % CI: 3.5 % to 10.2 %) than girls (0.3 % 95 % CI: 0.1 % to 1.3 %) and that smokeless tobacco usage was higher in rural regions (3.2 % 95 % CI: 1.9 % to 5.4 %) than in urban areas (1.8 % 95 % CI: 0.7 % to 4.4 %) (Table 2). Most adolescents (15–17 years), namely 85.2 % (95 % CI: 82.0 % to 88.0 %), acknowledged the detrimental effects of second-hand smoke. This recognition was stronger in urban areas (86.9 % (95 % CI: 82.3 % to 90.4 %) and among males (86.6 % (95 % CI: 82.1 % to 90.2 %)). Detailed information about age initiation of tobacco use among adolescents by area of residence and gender (Mean) is presented in supplementary file Table 5 and section 5.

### Alcohol use

**Fig. 3:**
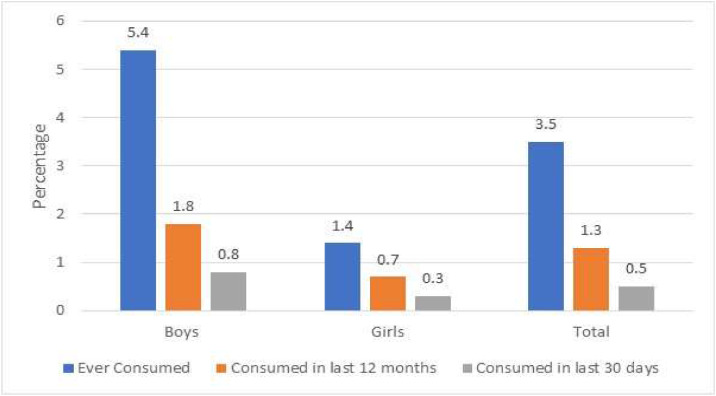
Alcohol use among adolescents by gender (Percentage).

The most popular kind of alcohol among those who had consumed alcohol in the 12 months prior to the survey, according to the results, was country liquor/some other sort (57.8 %), with a higher percentage of respondents from rural areas. Beer came in second place with 34.0 %. According to Fig. 3, 3.5 % of respondents had ever used alcohol in their lives, and 1.3 % and 0.5 %, respectively, had done so in the 12 months and 30 days prior to the survey. It was noted that boys tended to drink alcohol more frequently. According to where they lived, 1.4 % and 1.1 % of residents in rural and urban areas, respectively, and 3.6 % and 3.2 % of residents had ever used in the previous year prior to the survey. The results are presented in Table 3. The average age at which alcohol usage began was 13.4, and the average age gap between males (13.5 years) and girls (12.8 years) was over a year. It was noted that girls from rural regions were the youngest to begin (11.4 years). Detailed information about age (in years) of initiation of alcohol use among adolescents by area of residence and gender (Mean) is presented in supplementary file Table 4.

### Dietary behaviours

The frequency of consumption of food items at least once a week is 42.3 % (95 %CI 38.5–46.2), and at least once a month 38.5 % (95 %CI:34.5–42.6). The prevalence of cold drinks at least once a week is 18.2 %(95 %CI:15.4–21.5), and at least once a month 46 %(42.1–50.1), for fresh fruits at least once in a week 33.9 %(30.0–38.1), at least once in a month 34.1 %((30.5–37.9) and for high energy & protein drinks at least once in a week 6.5 %(4.4–9.6), at least once in a month 10.1 %(7.7–13.2).

**Fig. 4:**
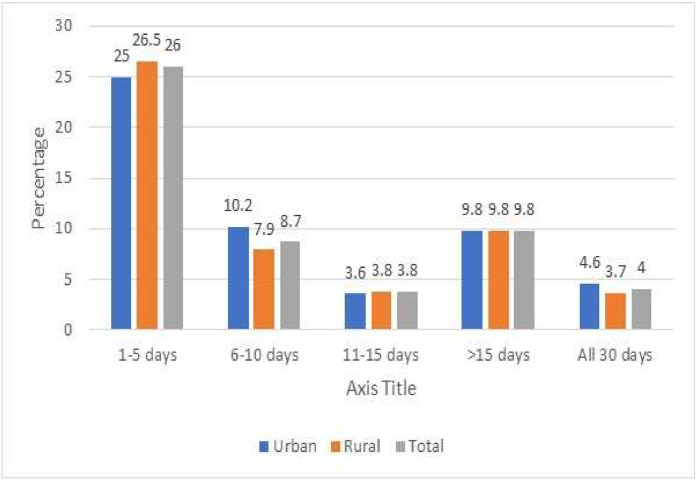
Adolescents aged 15–17 years who skipped breakfast in the past 30 days prior to the survey by area of residence (Percentage).

In the last thirty days prior to the survey, the mean number of days skipped for breakfast was 9.6. There was minimal variation in the number of extra days skipped by girls compared to boys, regardless of their residential area. In the last 30 days prior to the survey, 4.0 % missed breakfast every day and 48.3 % (45.8 % of boys and 50.9 % of girls) skipped breakfast at least once. The intake of chocolates and toffees (54.9 %) and chips and namkeen (52.1 %) was the highest, followed by sweets (32.1 %) and ice cream and milkshakes (20.9 %) on a daily and/or at least weekly basis. Fresh fruit, fruit juices, and salads with fruit chaat were consumed by 33.9 % and 27.8 % of respondents, respectively, on a daily and/or weekly basis. At least once a month, 20.4 % of people consume canned fruit juice. Instant noodles were consumed daily by 19.0 % of respondents, once a week by 30.1 %, and once a month by 30.1 % of respondents. Adolescents aged 15–17 years who skipped breakfast in the past 30 days preceding to the survey, by area of residence and gender (Percentage & 95 % CI) is presented in supplementary file Table 6. Fig. 4 presents the percentage of adolescents aged 15–17 years who skipped breakfast in the past 30 days, categorized by area of residence.

### Physical activity

The prevalence of physical activity levels among adolescents, for insufficient physical activity is 25.2(95 %CI: 22.2–28.5), and for sufficient physical activity 74.8(95 %CI:71.5–77.8). Time spent in physical activity per day at school, mean in minutes is 16.1 % (14.1–18.1) and Vigorous activity is 26.2 % (21.3–31.0), for Moderate activity is 78.3 % (72.8–83.8), for Leisure time activity is 11.8 % (9.8–13.7) and total minutes spent in physical activity is 104.5 % (96.1–112.9). Time (minutes) spent being sedentary in a day boys 335.1(281.3–389.0), girls 392.8(333.4–452.2), and 361.4(314.9–407.9). In terms of physical activity, 25.2 % of adolescents did not reach the WHO recommendations. The percentage of adolescents who engage in physical exercise is displayed in the supplementary file Fig. 2. Time (minutes) spent in physical activity per day by area of residence and gender (Mean) is presented in the supplementary file Table 1.

**Table 1:** Tobacco use, smokeless Tobacco use, and current daily tobacco uses among adolescents by area of residence and gender (Percentage).

15–17 Years	Urban			Rural			Total		
	Boys	Girls	Combined	Boys	Girls	Combined	Boys	Girls	Combined
**Tobacco use**	**95 % CI**								
Never User	89.9 (84.0–93.8)	99.7(97.9–100)	94.4(91.2–96.4)	87.2(82.3–90.9)	97.8(93.9–99.2)	92.3(89.4–94.5)	88.1(84.4–91.0)	98.3(95.7–99.4)	93.0(90.9–94.6)
Ever tobacco user/experimented	10.1(6.2–16.0)	0.3(0.04–2.1)	5.6(3.6–8.8)	12.8(9.1–17.7)	2.2(0.8–6.1)	7.7(5.5–10.6)	11.9(9.0–15.6)	1.7(0.6–4.3)	7.0(5.4–9.1)
**Smokeless tobacco uses among adolescents by area of residence and gender (Percentage)**									
**Smokeless tobacco use**	**95 % CI**								
Never User	94.1 (89.0–96.9)	99.7 (97.9–100)	96.7 (94.0–98.1)	90.6 (86.1–93.8)	98.1 (94.1–99.4)	94.3 (91.6–96.2)	91.8 (88.4–94.2)	98.6 (95.8–99.5)	95.0 (93.1–96.4)
Ever tobacco user/experimented	5.9 (3.1–11.0)	0.3 (0.04–2.1)	3.3 (1.9–6.0)	9.4 (6.2–13.9)	1.9 (0.6–5.9)	5.7 (3.8–8.4)	8.2 (5.8–11.6)	1.4 (0.5–4.2)	5.0 (3.6–6.9)
**Current daily tobacco uses among adolescents by area of residence and gender (Percentage)**									
Current daily tobacco use (either)	3.5 (1.4–8.3)	0.0 (0.0–0.0)	1.9 (0.8–4.5)	6.5 (3.9–10.7)	0.6 (0.2–1.8)	3.6 (2.3–5.8)	5.5 (3.5–8.6)	0.4 (0.1–1.3)	3.1 (2.0–4.7)
Only smoked tobacco	0.03 (0.004–0.2)	0.0 (0.0–0.0)	0.01 (0.002–0.1)	0.4 (0.1–2.4)	0.0 (0.0–0.0)	0.2 (0.03–1.3)	0.3 (0.1–1.6)	0.0 (0.0–0.0)	0.2 (0.03–0.8)
Only smokeless tobacco	3.2 (1.2–8.2)	0.0 (0.0–0.0)	1.8 (0.7–4.4)	6.0 (3.5–10.2)	0.3 (0.1–1.3)	3.2 (1.9–5.4)	5.1 (3.2–8.1)	0.2 (0.1–0.9)	2.8(1.7–4.3)
Both smoked and smokeless tobacco	0.2(0.03–1.3)	0.0(0.0–0.0)	0.1(0.02–0.7)	0.1(0.02–0.9)	0.3(0.04–1.7)	0.2(0.04–0.8)	0.2(0.04–0.6)	0.2(0.03–1.2)	0.2(0.05–0.5)

**Table 2:** Alcohol use among adolescents by area of residence and gender (Percentage).

15–17 Years	Urban			Rural			Total		
	Boys	Girls	Combined	Boys	Girls	Combined	Boys	Girls	Combined
		**95 % CI**							
Ever consumed	4.2 (2.0–8.6)	2.1 (0.7–5.9)	3.2 (1.5–6.6)	5.9 (3.3–10.6)	1.1 (0.4–3.5)	3.6 (2.1–6.1)	5.4 (3.3–8.6)	1.4 (0.6–3.1)	3.5 (2.2–5.4)
Consumed in last 12 months	1.4 (0.4–4.6)	0.6 (0.2–1.8)	1.1 (0.4–2.6)	2.0 (1.0–4.1)	0.7 (0.2–3.0)	1.4 (0.7–2.8)	1.8 (1.0–3.4)	0.7 (0.2–2.0)	1.3 (0.7–2.3)
Consumed in last 30 days	1.4 (0.4–4.6)	0.3 (0.1–1.3)	0.9 (0.3–2.5)	0.5 (0.1–2.1)	0.3 (0.04–1.7)	0.4 (0.1–1.2)	0.8 (0.3–2.0)	0.3 (0.1–1.0)	0.5 (0.2–1.2)
Alcohol use among adolescents*by type, area of residence, and gender (Percentage)									
Beer, lager, or stout	44.0(6.9–89.3)	31(3.8–83.5)	40.5(9.1–82.3)	(13.2–78.2)	0.0(0.0–0.0)	31.7(9.4–67.5)	42.9(16.5–74.1)	8.3(0.8–51.5)	34.0(13.4–63.1)
Wine/champagne	0.0(0.0–0.0)	15.6(1.4–70.4)	4.1(0.4–33.4)	0.0(0.0–0.0)	0.0(0.0–0.0)	0.0(0.0–0.0)	0.0(0.0–0.0)	4.2(0.4–33.4)	1.1(0.1–8.8)
Spirits, such as brandy/whisky/rum	2.1(0.2–22.1)	0.0(0.0–0.0)	1.5(0.1–15.1)	8.2(1.0–43.3)	0.0(0.0–0.0)	6.1(0.7–36.9)	6.6(1.0–34.2)	0.0(0.0–0.0)	4.9 (0.7–27.9)
Desi/some other type	53.9(8.3–93.8)	21.7(2.1–78.4)	45.3(8.7–87.8)	49.3(18.6–80.6)	100(100–100)	62.2(28.8–87.0)	50.5(22.3–78.4)	78.9(38.1–95.8)	57.8(30.2–81.3)
Ready-to-drink mixers	0.0(0.0–0.0)	0.0(0.0–0.0)	0.0(0.0–0.0)	0.0(0.0–0.0)	0.0(0.0–0.0)	0.0(0.0–0.0)	0.0(0.0–0.0)	0.0(0.0–0.0)	0.0(0.0–0.0)
Others	0.0(0.0–0.0)	31.7(3.4–85.9)	8.6(0.8–52.1)	0.0(0.0–0.0)	0.0(0.0–0.0)	0.0(0.0–0.0)	0.0(0.0–0.0)	8.6(0.8–52.1)	2.2(0.3–16.7)

⁎ Among those who consumed alcohol in the past 12 months.

### Physical measurements

Data was collected from survey participants who were willing to have their height (in centimetre’s) and weight (in kilograms) measured physically. The respondents ranged in age from 15 −17 years. The participants' height and weight were used to determine the Body Mass Index (BMI), which was computed using the following formula: BMI = Weight (Kg)/Height (m^2^). The respondents were divided into overweight and obese groups based on their BMI (as defined by the WHO). Obesity: >+2SD (corresponding to BMI 30.0 Kg/m^2^) and Overweight: >+1SD (corresponding to BMI 25.0 Kg/m^2^).

Both urban and rural areas had mean BMIs of 19.5 and 18.4 kg/m^2^, respectively. The mean BMI was 18.8 kg/m^2^. Measurements of height, weight, and BMI by area of residence and gender (Mean) are presented in the supplementary file Table 3.

#### Prevalence of overweight and obesity

In rural areas (3.6 percent overweight and 0.9 % obese) as well as urban areas (11.9 % overweight and 3.5 % obese). Boys made up 2.6 % of the obese while females made up 0.8 % of the overweight, according to gender. The prevalence of obesity and overweight in the 15–17-year-old age group was 1.8 % and 6.2 %, respectively.

## Discussion

An extensive nationwide survey known as the NNMS was conducted to establish baseline data for the national goals outlined in the NCD Monitoring Framework and Action Plan. Filling in the gaps in national data for this age range, it included information on metabolic and behavioural risk factors for adolescents (15–17 years old). In the world, tobacco usage poses a major threat to public health. It is becoming more and more like a pandemic, with severe illness, death, and disability. There is a lot of variance in consumption trends across all nations [22]. As per the latest assessment by the World Health Organization (WHO), the tobacco pandemic claimed the lives of 4.9 million individuals globally in 2000 due to nicotine addiction, and it continues to claim the lives of 5.4 million people annually [23]. The increased rates of alcohol and tobacco use (in any form) among males compared to girls were consistent with findings from earlier surveys, such as the Magnitude of Substance Use in India-2019, NFHS-4, and GATS-2 (National Institute on Drug Use) [24]. Additionally, the results of GATS-2 were consistent with a higher use of smokeless tobacco than smoked tobacco. 2.4 % of GATS-2 respondents reported using tobacco currently, compared to 3.1 % of NNMS respondents who reported using it daily [25]. The expected variances can be attributed to variations in the study design, sample approach, coverage, age groups chosen, weighting techniques, and questionnaires used. Upon closer inspection, we discovered that the GATS-2 survey's 15–17-year age group had a comparable prevalence of tobacco usage (3 %) to the present study [26]. According to the findings of the current study, chewing tobacco use was more common in men than in women, which is similar to the study done by Prajapati et al. [27,28]. According to the current study, boys and girls started smoking tobacco at mean ages of 14.2 and 14.4, respectively. According to Thakor et al., the mean age group of adolescents who ate betel nuts was 14.34 ± 1.83 years and 14.03 ± 1.41 years, in that order this is similar to the present study. The average age at which boys started smoking was 12.76 and 12.40 for girls, according to research by Meena et al. which is not consistent with the current study. Additional research revealed that in the majority of the world, tobacco use (cigarettes or other products) begins at age 13 or 14.

This study reveals key trends in the nutritional status and dietary behaviours of Indian adolescents aged 15–17 years, indicating early signs of health transition. A significant proportion exhibited excess body weight, with adolescents in urban areas more affected reflecting the influence of urbanization, changing food environments, and decreased physical activity. Gender differences in BMI categories suggest that cultural norms and awareness of healthy practices may influence weight outcomes differently among boys and girls. Unhealthy dietary patterns—such as low intake of fruits and vegetables and frequent consumption of processed foods—were prevalent, highlighting the potential for early development of noncommunicable diseases (NCDs). These patterns may be shaped by increased availability of energy-dense foods and reduced time spent preparing home-cooked meals, particularly in urban settings. Parental education and household income may also influence adolescents’ access to healthy food choices and opportunities for structured physical activity, contributing to inequalities in health behaviors across different socioeconomic groups. In addition, increased exposure to food marketing and digital advertisements, especially in urban areas, may further influence adolescents’ food preferences and promote the consumption of processed and unhealthy foods.

Breakfast skipping was highly prevalent, especially among girls. This behaviour may be associated with time constraints due to early school schedules, cultural expectations, or body image concerns. In some cases, adolescent girls may also have household responsibilities that interfere with regular meal consumption. Irregular eating patterns during adolescence can adversely affect metabolic health and overall growth during this formative stage.

Physical activity levels were suboptimal in a significant proportion of adolescents, with girls less likely than boys to meet recommended levels. This disparity may be influenced by social norms, safety concerns, and limited access to structured physical activity opportunities, especially in urban settings. The low average time spent on physical activity in school, as well as the dominance of moderate over vigorous activity, indicates that physical education is not sufficiently prioritized. Additionally, adolescents reported high levels of sedentary behaviour, including prolonged screen time, which may be replacing opportunities for physical or social engagement. This combination of low activity and high sedentary time presents an increased risk for early onset of lifestyle-related diseases. These findings highlight the need for school and community-based interventions that are culturally appropriate, gender-sensitive, and responsive to urban–rural differences.

The classification of overweight and obesity in this study was based on the WHO 2007 Growth Reference for children and adolescents aged 5–19 years [29]. This framework uses BMI-for-age >+1 SD to define overweight and >+2 SD for obesity. These cut-offs are internationally recognized and enable global comparability across adolescent health studies. The WHO 2007 reference has been widely adopted in Indian national surveys, including NFHS-5 [30] and the National Noncommunicable Disease Monitoring Survey (NNMS) 2017–18. Its use ensures alignment with both global standards and India's public health monitoring systems. Additionally, national guidelines—such as the Indian Academy of Pediatrics (IAP) 2015 growth charts [32] and the ICMR–NIN dietary and anthropometric guidelines [31]—reinforce the applicability of BMI-for-age in the Indian context. These references validate the interpretation of trends in adolescent nutritional status.

The study findings draw attention to a growing dual burden of malnutrition, where undernutrition persists in some segments while being overweight and obesity are increasing, particularly in urban areas. This trend reflects ongoing lifestyle transitions, including changes in diet and reduced physical activity. It highlights the urgent need for age-specific, culturally relevant interventions to prevent early onset of noncommunicable diseases.

Indian guidelines, such as those from the Indian Academy of Pediatrics (IAP) and the ICMR–NIN, reinforce the relevance of BMI-for-age as a key indicator of adolescent nutritional health. When interpreted against these national benchmarks, the findings draw attention to the dual burden of malnutrition—where overweight and obesity are rising even as undernutrition persists in some groups. These trends carry significant implications for adolescent health policy, suggesting a need for early screening, lifestyle education, and school-based interventions aimed at preventing long-term noncommunicable disease (NCD) risks. Although multivariate modelling was beyond the scope of this secondary data analysis, future research using the NMMS dataset or new primary data could apply adjusted models to identify independent determinants of adolescent overweight and obesity.

According to the current study, country liquor or another sort of alcohol was consumed in the preceding year in 57.8 % of cases. This result was similar to the findings of the Perdrix et al., study. The prevalence of alcohol use among teenagers in Seychelles was found to be 47.6 % in research conducted by Pengpid et al. [33]. Alcohol use was reported to be 61.1 % common in Seychelles, according to a study by Ma et al., that polled teenagers aged 13–15 [34]. The current study's findings, which were consistent with those of investigations by Assanangkornchai et al., Chaveepojnkamjorn et al., Georgie et al., and Pengpid & Peltzer, indicated that boys are more likely than girls to drink alcohol. Adolescents in four high schools in a Peri-urban district south of Johannesburg participated in a quantitative cross-sectional survey by Mohale and Mokwena, who found that alcohol consumption was 51 % among female adolescents. In contrast, alcohol use was more common in male adolescents (52 %). In sub-Saharan Africa (SSA), the pooled prevalence of alcohol consumption among adolescents has been estimated at 32.8 %; in contrast, in European countries, the prevalence among school-age adolescents has been estimated at 90 %. According to WHO estimates, 52.7 % of teenagers in the Americas and 69.5 % of those in Europe aged 15–19 drink alcohol.

## Limitations

Despite its valuable contributions, this study has several limitations that should be acknowledged. First, the focus on adolescents aged 15–17 years limits the generalizability of findings to the broader adolescent population (10–19 years). Second, behavioral risk factors—such as tobacco and alcohol use, dietary patterns, and physical activity—were self-reported, introducing potential recall and social desirability biases. Third, due to the use of secondary data from the NNMS 2017–18 survey, individual-level variables were not accessible, which restricted the ability to perform multivariate analyses to identify dominant determinants of dietary behavior and physical activity. The cross-sectional design further restricts the ability to draw causal inferences or track changes in risk factors over time. Additionally, the study did not capture key determinants such as mental health status, socioeconomic background, stress, and sleep behaviours, all of which are increasingly recognized as important contributors to non-communicable disease risk among adolescents. Physical activity was also assessed through self-reporting, which may not fully reflect the intensity or diversity of adolescent movement patterns. Nevertheless, these limitations do not diminish the significance of the study as a foundational effort to generate nationally representative data on adolescent NCD risk factors, which can inform future research and public health strategies. Future follow-up studies may benefit from longitudinal designs to track behavior change over time, and from the use of objective measurement tools—such as accelerometers or wearable devices—to validate self-reported physical activity.

## Ethics statements

Not Applicable. This study is based on secondary data from the National Noncommunicable Disease Monitoring Survey (NNMS), 2017–18, conducted by the Indian Council of Medical Research (ICMR) and the Ministry of Health and Family Welfare, Government of India. The original survey received ethical approval from the ICMR Central Ethics Committee. The dataset is anonymized and publicly accessible to researchers upon request. Therefore, no additional ethical approval was required for this secondary data analysis.

## Declaration of competing interest

The authors declare that they have no known competing financial interests or personal relationships that could have appeared to influence the work reported in this paper.

## Data Availability

Data will be made available on request.

## Appendix Supplementary materials

Image, application 1
