# Supplementary material for: A national survey-based method for monitoring noncommunicable disease risk factors among adolescents aged 15–17 years across India
Source: MethodsX. 2025 Jul 24;15:103535. doi: 10.1016/j.mex.2025.103535 (PMC12337017; doi:10.1016/j.mex.2025.103535)
Supplement: Supplementary file 1 [file mmc1.docx]

## **Supplementary Material**

The three-level stratification and sampling frame in urban areas are given in Figure 1.


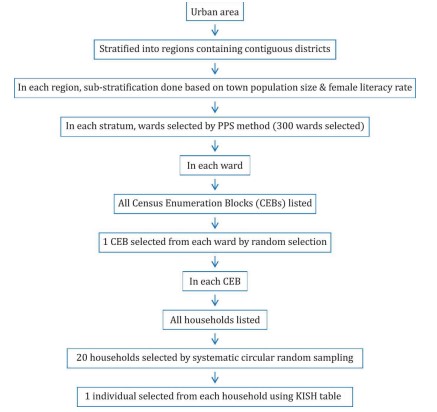


**Figure 1:** The three-level stratification and sampling frame in urban areas

Table 1. Time (minutes) spent in physical activity per day by area of residence and gender (Mean)

| **15-17 years** | **Urban** | **Rural** | **Boys** | **Girls** | **Total** |
| --- | --- | --- | --- | --- | --- |
| Vigorous activity | 11.1 | 33.1 | 31.9 | 19.9 | 26.2 |
| Moderate Activity | 59.3 | 87.1 | 67.8 | 89.9 | 78.3 |
| Total minutes spent in physical activity | 70.4 | 120.2 | 99.7 | 109.8 | 104.5 |
| Leisure time activity | 14.3 | 10.6 | 17.2 | 5.9 | 11.8 |

Table 2: List of indicators, definitions and instruments used in the National NCD monitoring Survey (NNMS) 2017-18.

| **Indicator** | **Instruments** | **Definition** |
| --- | --- | --- |
| Current tobacco use | Questionnaire | Adults who used any form of tobacco (smoke and/or smokeless) in the last 12 months preceding the survey. |
| Current daily smoked tobacco use | Questionnaire | Adults who used any smoked tobacco products such as bidis, cigarettes, cigars, chillum, pipes, hookah or any other local smoked tobacco products daily in the last 12 months preceding the survey. |
| Current daily smokeless tobacco use | Questionnaire | Adults who used any smokeless tobacco products such as chewing tobacco, paan with tobacco, tobacco snuff by mouth or nose daily in the last 12 months preceding the survey. |
| Second hand tobacco smoke exposure at home | Questionnaire | Adults who reported being exposed to tobacco smoke on one or more than one occasion due to someone smoking tobacco close by at home in the past 30 days. |
| Current alcohol use | Questionnaire | Adults who consumed any form of alcohol (such as beer, wine, whisky, locally prepared alcohol, etc) in the last 12 months preceding the survey. |
| Heavy episodic drinking | Questionnaire | Adults those who engaged in consuming ≥6 standard drinks (equivalent to 60 grams of pure alcohol or ethanol) in a single drinking occasion over the past 30 days. |
| Insufficient physical activity | Questionnaire | Adults who engaged in <150 minutes of moderate – intensity physical activity per week OR <75 minutes of vigorous – intensity physical activity per week OR an equivalent combination of moderate- and vigorous- intensity physical activity accumulating <600 MET – minutes per week. |
| Overweight and obesity | Weight: Digital weighing scale (SECA 803) and Height: Portable stadiometer (SECA 213) | Overweight: BMI: 25.0–29.9 Kg/m^2^; Obesity: BMI≥ 30.0 Kg/m^2^ as per WHO cut-off. |
| Central Obesity | Waist circumference measured using Measuring or tension tape (SECA, 201) | Waist circumference of ≥90cm in males and ≥80cm in females (as per South Asia Pacific Guidelines). |
| Raised blood pressure | 1) Measurement of blood pressure using Automatic blood pressure machine (OMRON HEM–7120, Omron corporation, Kyoto, Japan). | Adults with a systolic blood pressure ≥140 mmHg and/or diastolic blood pressure ≥90 mmHg including those on medication for raised blood pressure. |
|  | 2) Questionnaire - Self reported medical history of raised blood pressure |  |
| Raised fasting blood glucose | 1) Fasting blood glucose using Glucometer (Gluco spark, Sensa core, Telangana, India) | Adults with fasting blood glucose value ≥126 mg/dl including those on medication for raised blood glucose. |
|  | 2) Questionnaire - Self reported medical history of raised blood glucose |  |

**Table 3:** Measurements of height, weight, and BMI by area of residence and gender (Mean)

| **15-17 years** | **Urban** | **Rural** | **Boys** | **Girls** | **Total** |
| --- | --- | --- | --- | --- | --- |
| Height(cm) | 159.2 | 157.2 | 162.9 | 152.4 | 157.9 |
| Weight (Kg) | 49.5 | 45.5 | 49.0 | 44.4 | 46.8 |
| BMI(Kg/m^2^) | 19.5 | 18.4 | 18.5 | 19.1 | 18.8 |


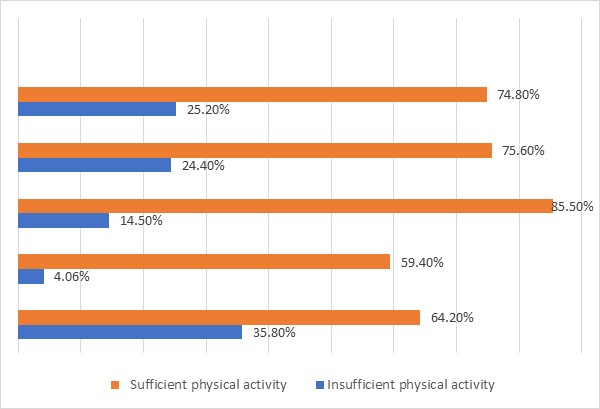


**Figure 2:**  Physical activity levels among adolescents (Percentage)

**Table 4**: Age (in years) of initiation of alcohol use among adolescents (15-17 years) by area of residence and gender (Mean)

| **15-17 years** | **Urban** | | | | **Rural** | | | Total | | |
| --- | --- | --- | --- | --- | --- | --- | --- | --- | --- | --- |
|  | **Boys** | **Girls** | **Combined** | **Boys** | | **Girls** | **Combined** | **Boys** | **Girls** | **Combined** |
|  | (**95% CI)** | | | | | | | | | |
| Age Initiation | 14.0 | 14.5 | 14.1 | 13.4 | | 11.4 | 13.1 | 13.5 | 12.8 | 13.4 |
|  | (12.1-15.8) | (13.1-16.0) | (12.8-15.4) | (12.3-14.5) | | (7.3-15.5) | (12.0-14.2) | (12.6-14.5) | (10.2-15.4) | (12.5-14.3) |

**Table 5**: Age (in years) of initiation* of tobacco use among adolescents (15-17 years) by area of residence and gender (Mean)

| **15-17 years** | **Urban** | | | | **Rural** | | | Total | | |
| --- | --- | --- | --- | --- | --- | --- | --- | --- | --- | --- |
|  | **Boys** | **Girls** | **Combined** | **Boys** | | **Girls** | **Combined** | **Boys** | **Girls** | **Combined** |
|  | (**95% CI)** | | | | | | | | | |
| Tobacco | 15(14.3-15.8) | 17(17.0-17.0) | 15.1(14.3-15.9) | 13.9(13.0-14.8) | | 14.1(12.9-15.2) | 13.9 (13.1-14.7) | 14.2(13.5-14.9) | 14.4(13.2-15.5) | 14.2(13.5-14.9) |
| **Smoked tobacco** | 15.1(14.0-16.2) | 0.0(0.0-0.0) | 15.1(14.0-16.2) | 14.7 (14.2-15.1) | | 14.7 (14.0-15.5) | 14.7(14.3-15.1) | 14.8(14.3-15.3 | 14.7(14.0-15.5) | 14.8(14.3-15.3) |
| **Smokeless tobacco** | 14.9(14.2-15.6) | 17.0(17.0-17.0) | 15(14.3-15.7) | 13.7(12.6-14.8) | | 13.5(12.2-14.8) | 13.7(12.7-14.7) | 14.0(13.0-14.9) | 14.0(12.5-15.5) | 14.0(13.1-14.8) |

*Among ever tried/experimented tobacco users

**Table 6**: Adolescents aged 15-17 years who skipped breakfast in the past 30 days preceding to the survey, by area of residence and gender (Percentage)

| **15-17 years** | **Urban** | | | | **Rural** | | | Total | | |
| --- | --- | --- | --- | --- | --- | --- | --- | --- | --- | --- |
|  | **Boys** | **Girls** | **Combined** | **Boys** | | **Girls** | **Combined** | **Boys** | **Girls** | **Combined** |
|  | **(95% CI)** | | | | | | | | | |
| Never skipped  breakfast | 56.3(47.5-64.8) | 45.2(37.3-53.5) | 51.3(44.9-57.7) | 53.1(46.5-59.6) | | 50.7(44.2-57.1) | 51.9(47.1-56.7) | 54.2(48.8-59.4) | 49.1(43.9-54.2) | 51.7(47.8-55.6) |
| Skipped breakfast on any one day | 43.7(35.2-52.5) | 54.8(46.5-62.7) | 48.7(42.3-55.1) | 46.9(40.4-53.5) | | 49.3(42.9-55.8) | 48.1(43.3-52.9) | 45.8(40.6-51.2) | 50.9(45.8-56.1) | 48.3(44.4-52.2 |
| **Number of days breakfast was skipped prior to the survey** |  | | | | | | | | | |
| 1 – 5 days | 23.9(17.9-31.2) | 26.4(20.5-33.2) | 25.0(20.5-30.2) | 26.2(20.0-33.6) | | 26.7(21.1-33.1) | 26.5(22.0-31.4) | 25.5(20.7-30.9) | 26.6(22.2-31.4) | 26.0(22.6-29.7) |
| 6 – 10 days | 10.0(6.1-15.9) | 10.4(6.4-16.5) | 10.2(6.9-14.8) | 8.0(5.1-12.4) | | 7.9(5.3-11.6) | 7.9(5.8-10.7) | 8.7(6.2-12.0) | 8.6(6.3-11.7) | 8.7(6.8-11.0) |
| 11 – 15 days | 2.9(1.3-6.5) | 4.5(2.5-8.2) | 3.6(2.1-6.1) | 3.2(1.8-5.8) | | 4.5(2.4-8.3) | 3.8(2.5-5.8) | 3.1(1.9-5.0) | 4.5(2.8-7.2) | 3.8(2.7-5.3) |
| >15 days | 6.9(3.0-15.1) | 13.4(8.8-19.8) | 9.8(6.0-15.8) | 9.4(5.8-15.1) | | 10.3(7.0-14.9) | 9.8(7.0-13.7) | 8.6(5.6-12.9) | 11.2(8.4-14.8) | 9.8(7.4-12.9) |
| All 30 days | 4.4(1.2-14.5) | 4.9(2.3-9.9) | 4.6(1.8-11.4) | 3.5(1.7-7.2) | | 3.8(2.1-6.8) | 3.7(2.2-6.0) | 3.8(1.9-7.3) | 4.1(2.6-6.5) | 4.0(2.5-6.3) |
